# Supplementary material for: Comparative multi-omics analyses reveal differential expression of key genes relevant for parasitism between non-encapsulated and encapsulated Trichinella
Source: Commun Biol. 2021 Jan 29;4:134. doi: 10.1038/s42003-021-01650-z (PMC7846577; doi:10.1038/s42003-021-01650-z)
Supplement: Supplementary file 2 — Supplementary Information [file 42003_2021_1650_MOESM2_ESM.pdf]

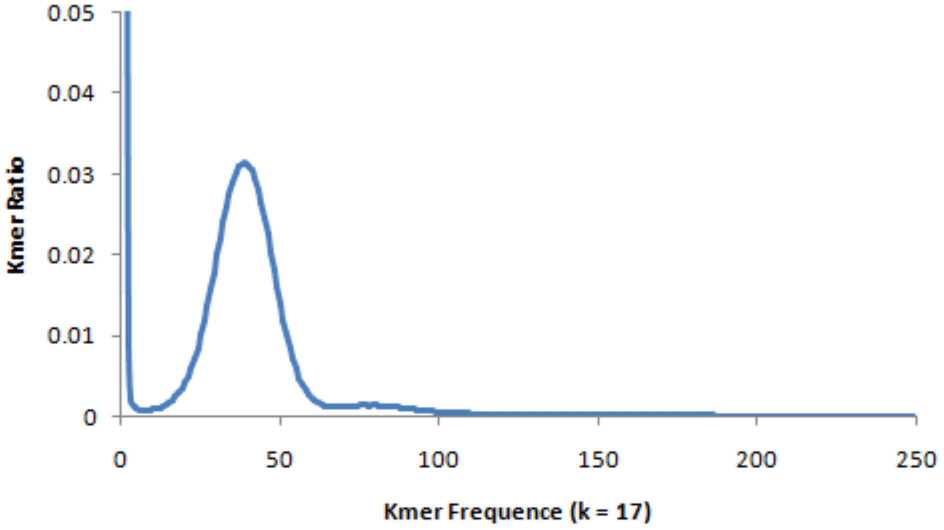

Supplementary Figure 1. The *T. pseudospiralis* genome size estimated by 17-mer analysis. Jellyfish (v1.1.12), which is a tool for fast, memory-efficient *k*-mer counters, was applied for calculating occurrence of 17-mers from high-quality Illumina reads.

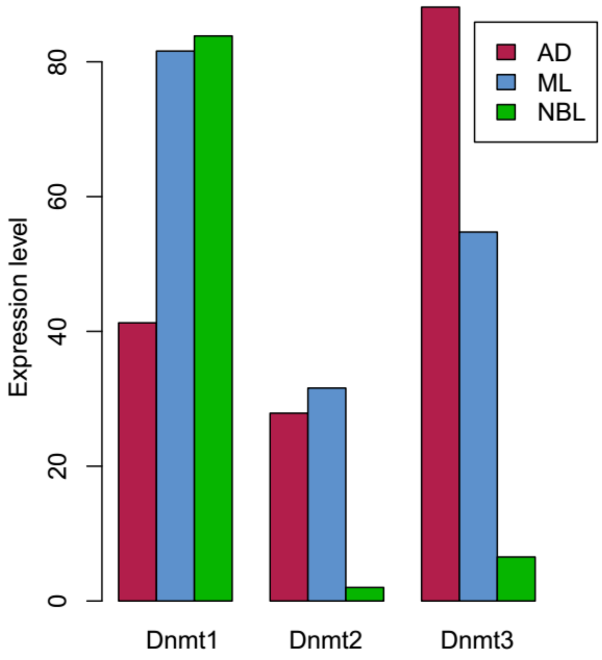

Supplementary Figure 2. Expression levels of DNMT genes revealed by transcriptome analysis.

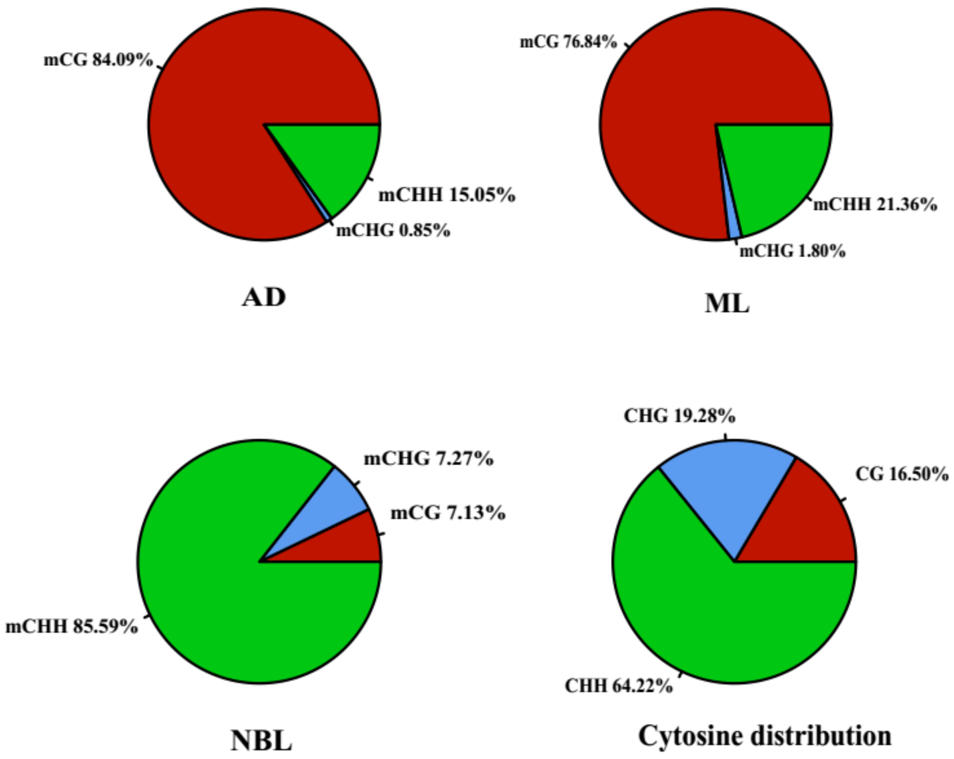

Supplementary Figure 3. The fraction of mCs identified in each sequence context in comparison with the fraction of all Cs in each sequence context in the *T. pseudospiralis* genome. All three life stages are included.

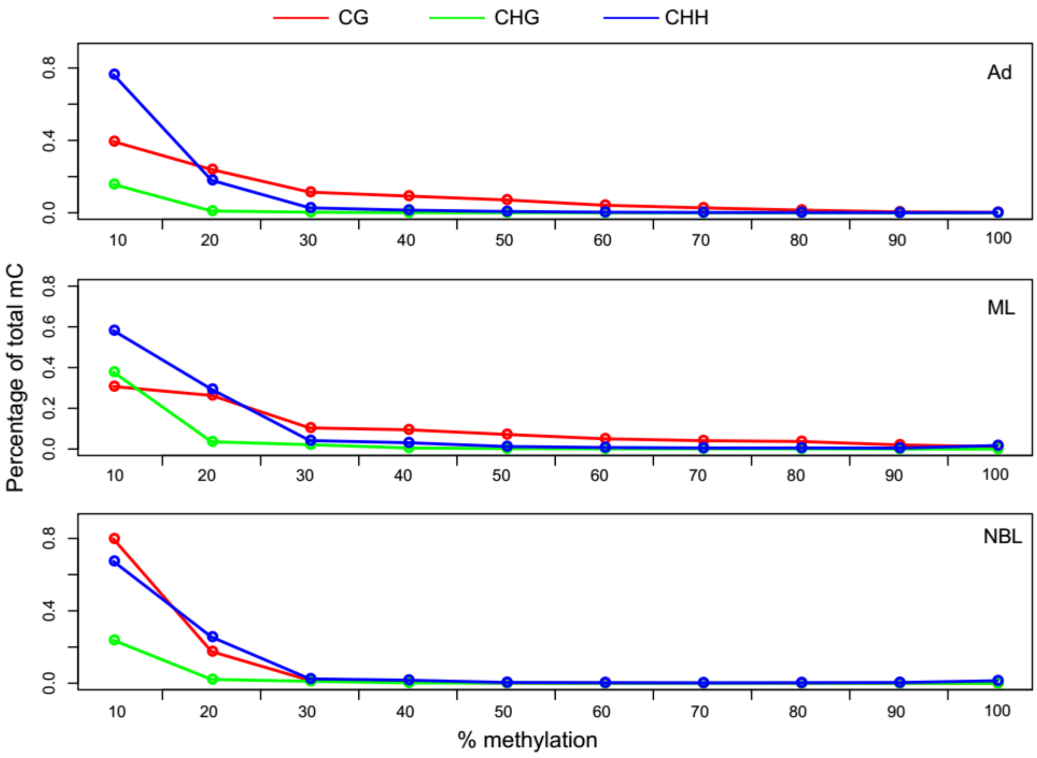

Supplementary Figure 4. Distribution of mCs (y-axis) across the percentage of methylation levels (x-axis).

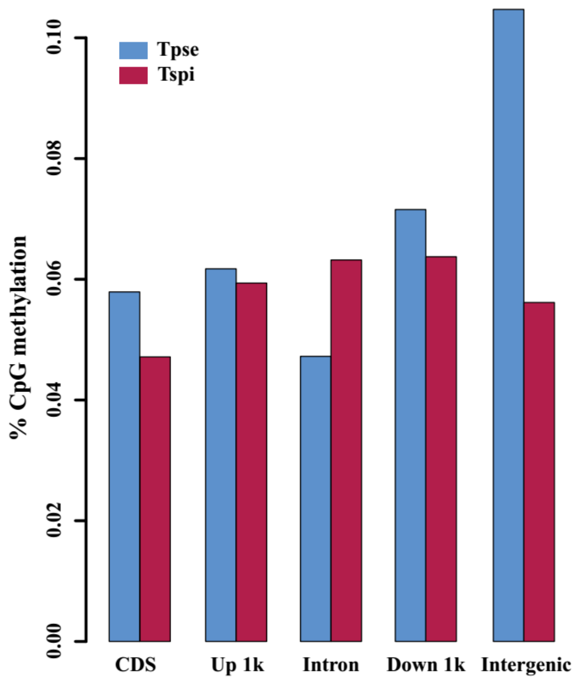

Supplementary Figure 5. Methylation differences in genic and intergenic regions of *T. pseudospiralis* and *T. spiralis* (distribution of common mCG sites with depth ≥ 5x). Upstream/downstream regions are defined as 1 kb starting from the transcriptional start site without including the intergenic regions.

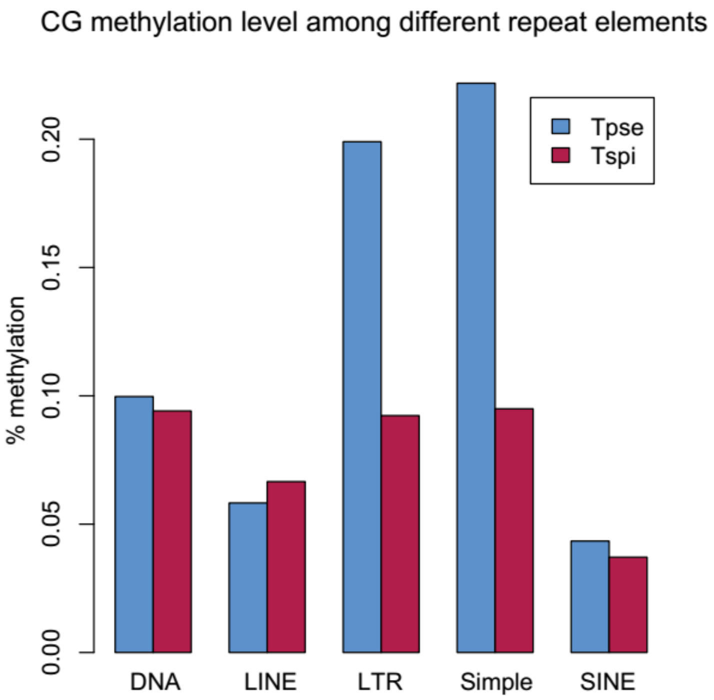

Supplementary Figure 6. Comparison of methylation levels among different repetitive elements in *T. pseudospiralis* and *T. spiralis* genomes.

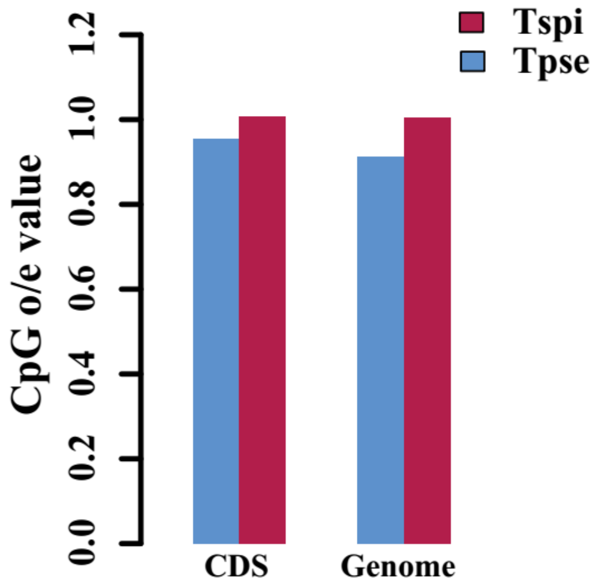

Supplementary Figure 7. Compariosn of $CpG_{o/e}$ value across whole genome and coding regions in *T. pseudospiralis* and *T. spiralis*.

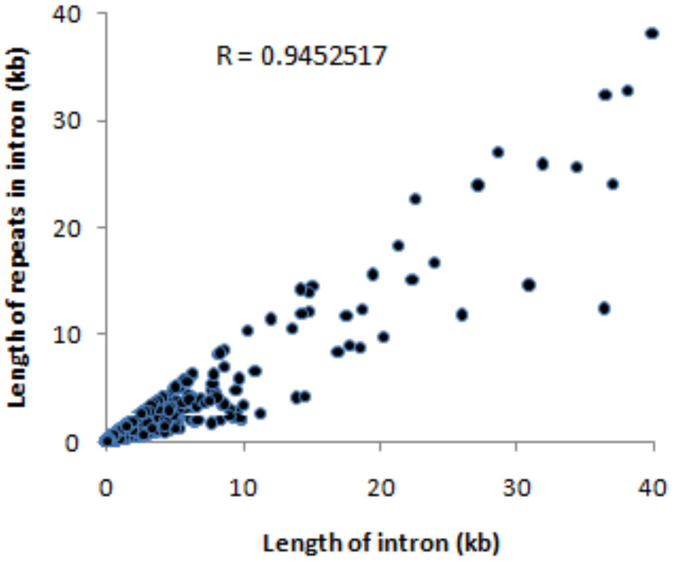

Supplementary Figure 8. Pearson's correlation analysis between intron and intronic TE lengths for *T. pseudospiralis*.

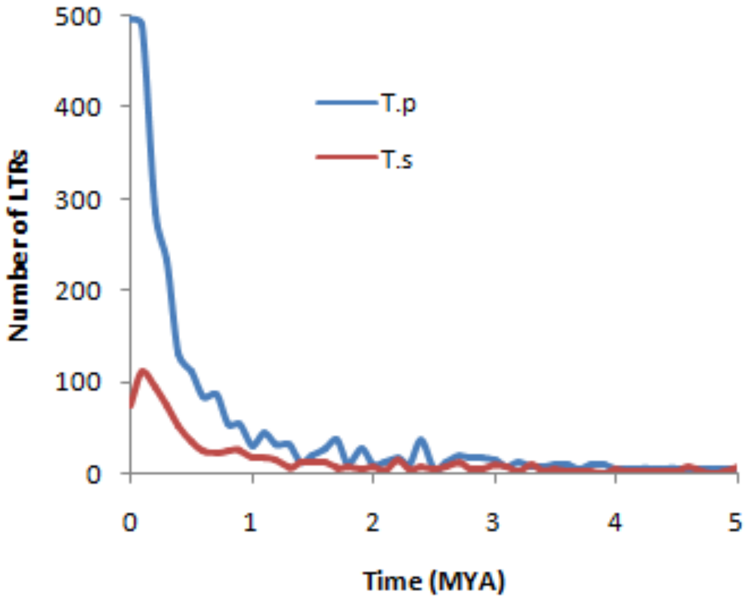

Supplementary Figure 9. Distribution of divergence times for the complete long terminal repeats (LTRs) for *T. pseudospiralis*, in million years ago (mya).

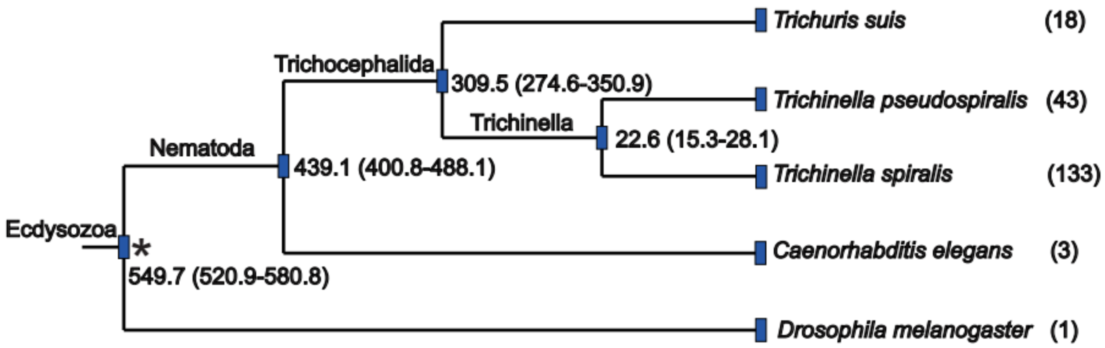

Supplementary Figure 10. Species tree of five species used in this study. Divergence time is given in mya. Numbers in brackets represent the estimated DNase II gene number.

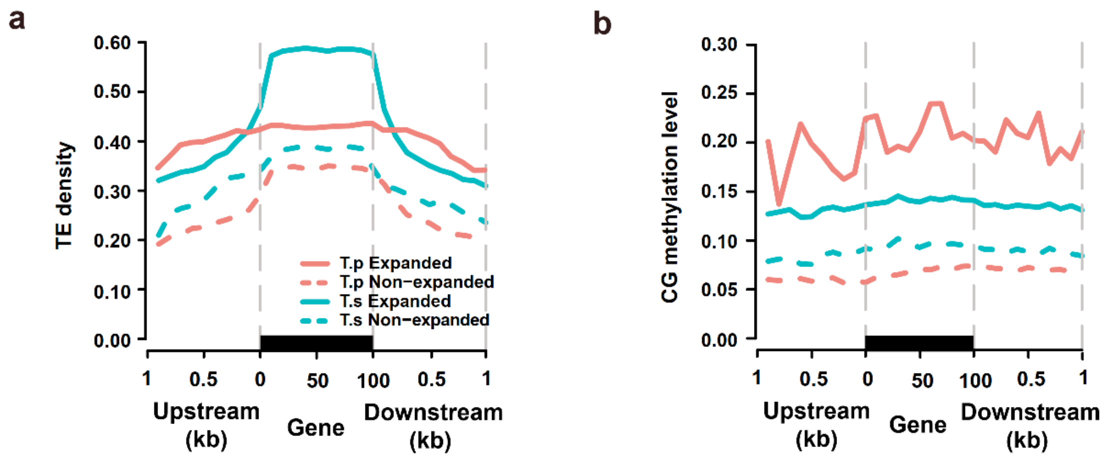

Supplementary Figure 11. Comparison of expanded and non-expanded gene families with regard to TE density and DNA methylation level. (a) Comparison of average density of transposon element (y-axis) between expanded and non-expanded gene families containing TEs around genes and their flanking regions (x-axis). Two-kilobase regions upstream and downstream of each gene were divided into 20 intervals, and so were genic regions. **(b)** Comparison of methylation levels (y-axis) between expanded and non-expanded gene families containing TEs around gene and their flanking regions (x-axis). Two-kilobase regions upstream and downstream of each gene were divided into 20 intervals, and so were genic regions.

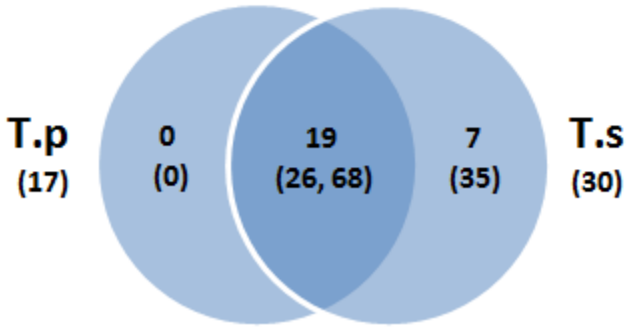

Supplementary Figure 12. Comparison of gene family size of DNase II superfamily between *T. pseudospiralis* and *T. spiralis*. Numbers in and out the parentheses represent gene and gene family numbers.

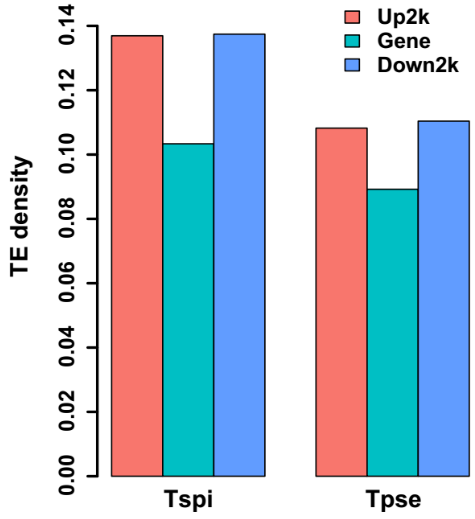

Supplementary Figure 13. Comparison of average density of transposon element (y-axis) across the whole genome and their two-kilobase flanking regions upstream and downstream in *T. spiralis* and *T. pseudospiralis* (x-axis).

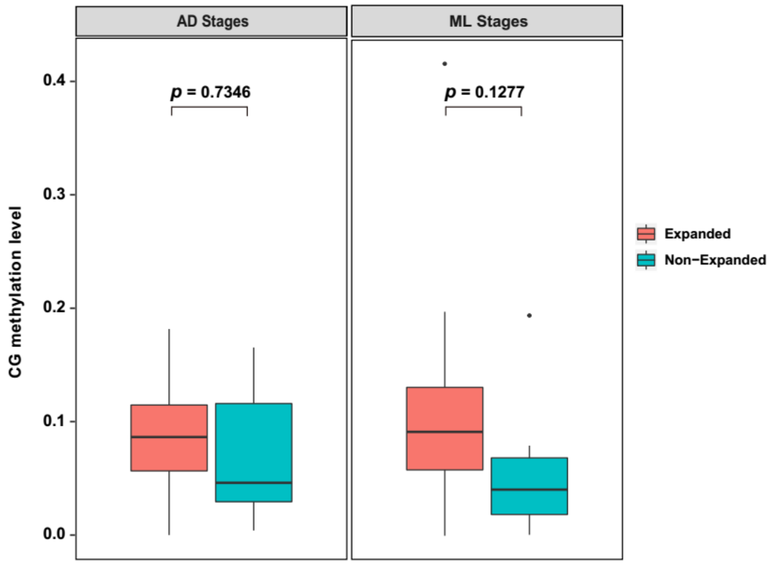

Supplementary Figure 14. Comparison of methylation level between expanded and non-expanded DNase II promoter regions in Ad (left) and ML (right) stages in *T. spiralis*. A Mann-Whitney *U* test was applied to the pairwise comparison.

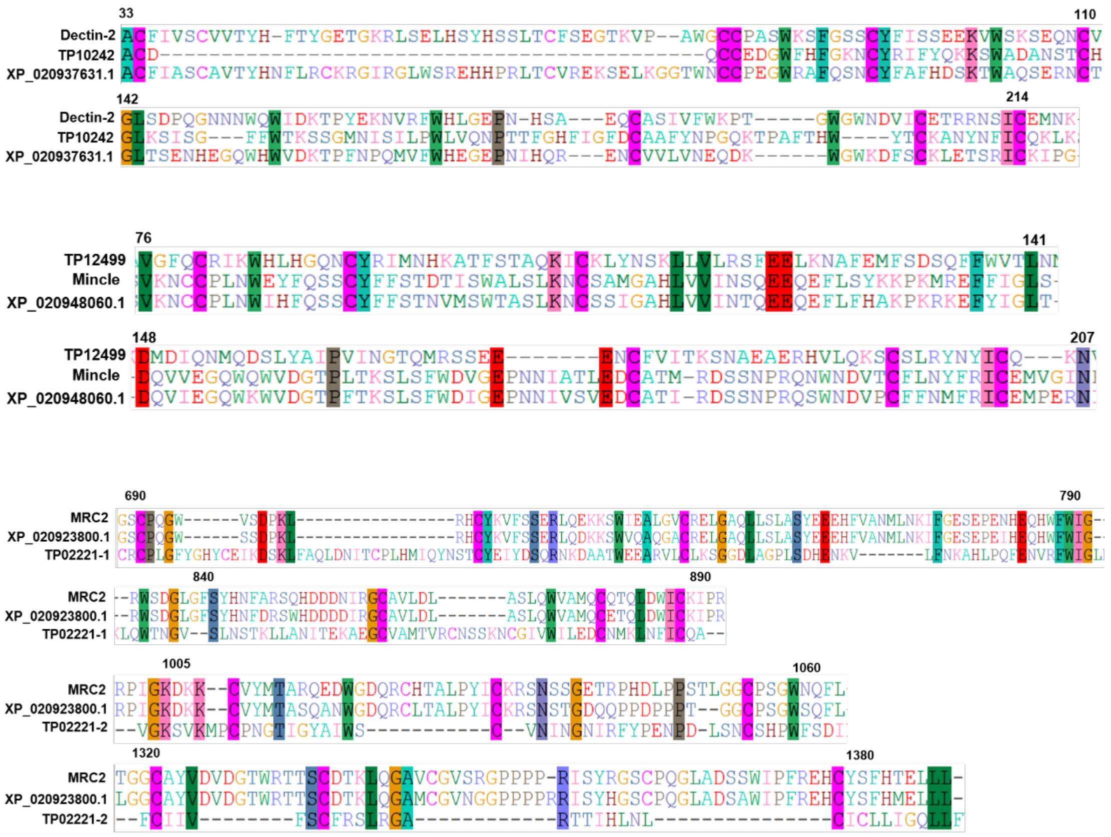

Supplementary Figure 15. Sequence alignment of C-type lectins with its homology of mammalian host. Conserved cysteine residues are highlighted in purple. The Dectin-2 represents the homologous protein in mammalian host *Homo sapiens*. XP_020937631.1 represents the homologous protein in *Sus scrofa*. TP02221-1 represents the first lectin domain, and TP02221-2 represents the second lectin domain in TP02221.

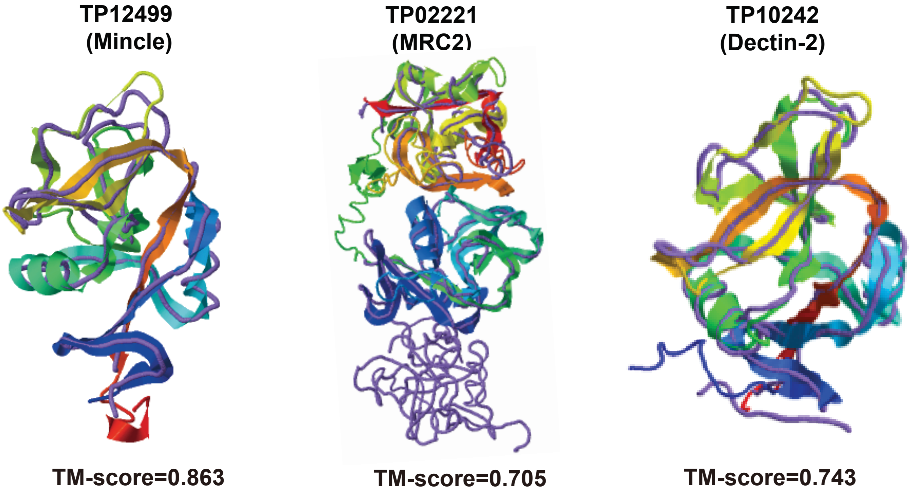

Supplementary Figure 16. Structure model of TP12499, TP02221 and TP10242. Structural model of the C-type lectin domains is shown in cartoon, while the structural analog in the PDB library is displayed using backbone trace and colored in purple (as identified by TM-align). The protein model is performed by I-TASSER. The TM-score value scales the structural similarity of the two structures.

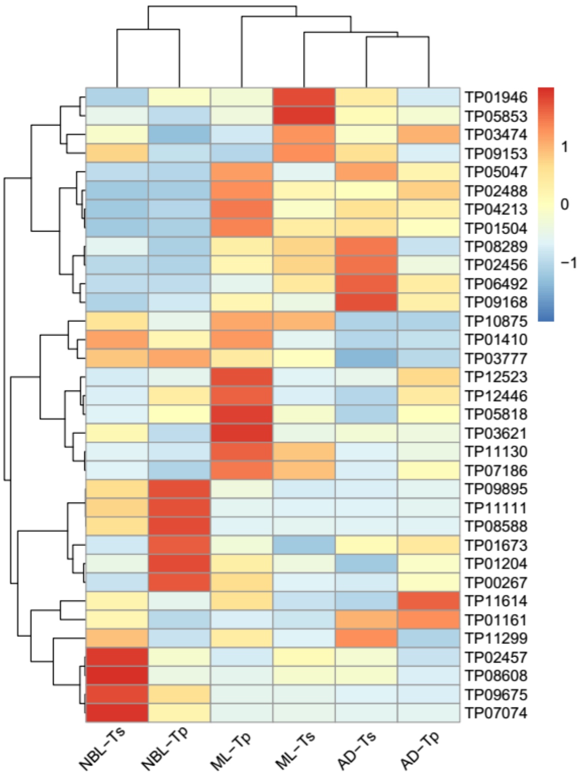

Supplementary Figure 17. Heatmap of 34 DEGs between *T. pseudospiralis* and *T. spiralis*.

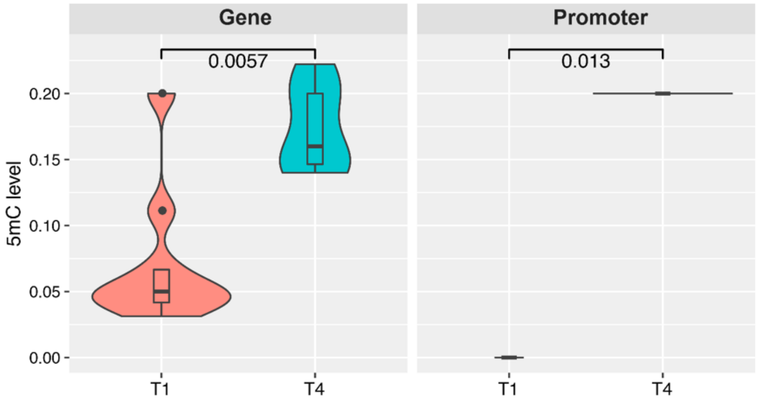

Supplementary Figure 18. Comparison of methylation level of TP12446 between *T. pseudospiralis* (T4) and *T. spiralis* (T1) in gene-body and promoter regions in ML stage. A Mann-Whitney *U* test was applied to the pairwise comparison.

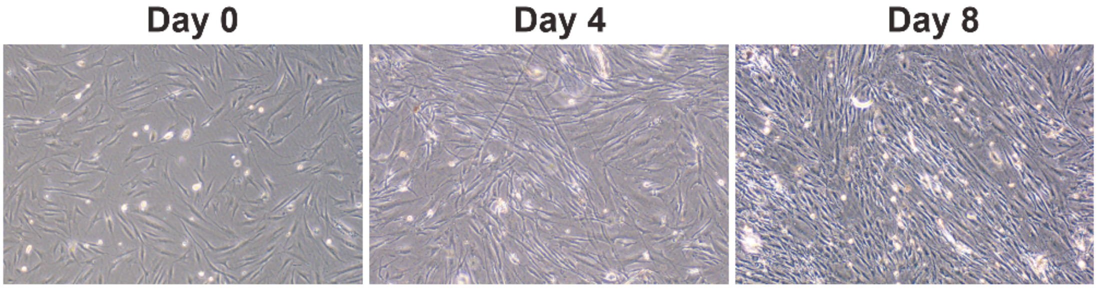

Supplementary Figure 19. Undifferentiated myoblasts C2C12 and terminally differentiated myotubes at 4 or 8 days.

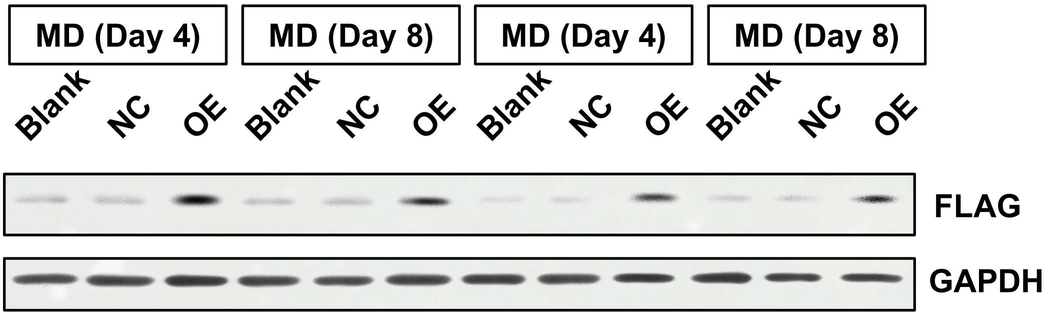

Supplementary Figure 20. Protein expression levels of TP12446 were assessed by western blot from myotubes cultured for 4 or 8 days. The three transgenic mouse cell lines were: 1) normal cell (Blank), 2) an empty lentiviral vector PSE-CMV-NC (NC), 3) a lentiviral vector PSE-CMV-TP12446 with overexpression of TP12466 (OE).

Supplementary Table 1. Summary of sequencing data for the *T. pseudospiralis* genome.

Sequence data	Stage	Sequencing method	Insert size (bp)	Average length (bp)	Raw data	Clean data	Sequence depth (X)
Genomic DNA	ML	Illumina	209	125	8.01 Gb	6.90 Gb	98
	ML	Pacbio	20,000	~2,991	18.05 Gb	9.93 Gb	144

Supplementary Table 2. Assessment of sequence coverage of *T. pseudospiralis* genome assembly using RNA-seq data.

Type	Insert size (bp)	Read length (bp)	Stage	Mapped reads	Mapped bases (bp)	Mapping ratio (%)
RNA-seq	~200	150; 150	Ad	16,177,830	4,853,344,317	95.11
			ML	14,686,278	4,405,878,642	94.60
			NBL	17,632,702	5,289,805,326	93.87

Supplementary Table 3. Assessment of sequence coverage of the *T. pseudospiralis* genome assembly using ESTs. EST sequences are aligned on the assembled genome using BLAT with an identify cutoff of 95%. The ESTs were retrieved from NCBI dbEST.

Dataset	Number	Total length (bp)	Covered by assembly (%)	>90% of sequence covered by one scaffold		>50% of sequence covered by one scaffold	
				Number	Percent	Number	Percent
**AD stage**							
>0bp	18,904	9,653,238	97.43	18,074	95.61	18,840	99.66
>200bp	18,017	9,519,510	97.51	17,445	96.83	17,977	99.78
>500bp	11,132	6,798,390	97.71	10,874	97.68	11,113	99.83
**ML stage**							
>0bp	18,270	9,448,895	95.74	17,008	93.09	18,111	99.13
>200bp	17,553	9,340,796	95.81	16,567	94.38	17,412	99.20
>500bp	11,215	6,836,388	96.07	10,945	97.59	11,129	99.23
**NBL stage**							
>0bp	17,311	6,648,836	90.55	13,375	77.26	16,261	93.93
>200bp	15,405	6,362,589	91.03	12,428	80.68	14,625	94.94
>500bp	3,623	2,033,888	91.55	3,228	89.10	3,426	94.56

Supplementary Table 4. Comparison of genome features between the two assembly versions of *T. pseudospiralis* genome.

Term	T4_ISS13_R	T4_ISS13_r1.0
**Overall**		
17-mer estimated genome size (in Mb)	69.79	69.78
Total scaffolded assembly size (Mb); total scaffolds	68.90; 2,746	49.16; 7,221
Total scaffolds of > 2kb: length (Mb); no. of scaffold	68.71; 2,631	46.64; 406
Largest scaffold (Mb)	3.73	1.95
Gaps, combined length (kb)	29.69	905.3
N50 in kb of scaffolds; count > N50 length	208.90; 68	235.43; 51
N90 in kb of scaffolds; count > N90 length	7.68; 1,231	60.44; 206
N50 in kb of contigs; count > N50 length	174.42; 75	122.06; 112
N90 in kb of contigs; count > N90 length	7.56; 1,321	13.81; 415
Repetitive sequences (%)	40.28%	18.01%
BUSCO genes (%)	88.6%	87.2%
**Protein coding regions**		
Number of gene models	12,682	12,656
Gene density (genes per Mb)	184	257
Exonic proportion, including introns (bp)	29,554,476	28,674,747
Mean/Median gene size (bp)	2330.43/1077	2265.70/1089
Mean/Median CDS size (bp)	1039.25/627	1045.78/523.5
Number of exons	69,966	76,053
Number of bp included in exons	13,179,752	13,235,355
Mean/Median exon size (bp)	188.37/137	174.03/124
Mean number of exons per gene	5.52	6.01
Number of introns	57,284	63,397
Number of bp included in introns	16,374,723	15,439,393
Mean/Median intron size (bp)	285.85/76	243.54/76
Overall G + C content (%)	31.49	32.60
Exons, G + C content (%)	42.25	42.33
Introns, G + C content (%)	28.90	27.45
Intergenic regions, G + C content (%)	29.48	30.32
Number of predicted excretory-secretory protein	471	443
Number of expressed genes (RPKM > 10)	7,170	5,874

Supplementary Table 5. General statistics of predicted protein-coding genes.

Gene set		Number	Average length of transcribed region (bp)	Average length of CDS (bp)	Average length of exon (bp)	Exons per gene	Average Length of intron (bp)
ab initio	SNAP	16,486	2,126	920	138	6.7	214.8
	Augustus	7,545	3,220	1,524	198	7.7	255.1
	GlimmerHMM	19,654	1,567	740	199	3.7	305.7
Homology based	*A. suum*	3,395	1,936	1,140	189	6.0	161.1
	B. malayi	2,749	1,902	1,138	190	6.0	155.7
	C. elegans	2,822	2,163	1,264	191	6.6	161.9
	T. spiralis	11,688	1,799	1,019	185	5.5	175.2
	M. incognita	2,062	1,835	1,074	186	5.8	160.9
	T. suis	4,192	2,662	1,468	205	7.1	196.5
	H. sapiens	2,636	2,074	1,243	186	6.7	148.4
	Uniprot	2,664	2,077	1,234	186	6.6	151.9
Transcriptome aligment	RNA-seq	11,396	4,729	2,683	343	7.8	301.9
GLEAN		12,682	2,330	1,039	188	5.5	285.9

Supplementary Table 6. Number of genes with homologous or functional classification by each annotated method.

	Database	Number	Percent (%)
Annotated		11,308	89.17
	InterPro	6,696	52.80
	GO	4,928	38.86
	KEGG	5,260	41.48
	Swissprot	6,275	49.48
	TrEMBL	9,833	77.54
	NR	10,912	86.04
	NT	10,733	84.63
Unanotated		1,374	10.83
Total		12,682	100

Supplementary Table 7. Summary of whole genome bisulfite sequencing data.

Life stage	Raw reads	Raw bases	Clean reads	Clean bases	Mapped reads	Mapped ratio (%)	Duplication ratio (%)	Conversion ratio (%)
**AD**	26,900,038	4,035,005,700	24,705,792	3,558,281,529	21,367,757	86.49	9.17	99.69
**ML**	23,616,128	3,542,419,200	21,220,806	3,068,794,243	20,117,449	94.80	9.96	99.73
**NBL**	22,369,224	3,355,383,600	20,246,560	2,928,874,108	19,409,575	95.87	10.15	99.36

Supplementary Table 8. Comparison of repeat content between *T. pseudospiralis* and *T. spiralis*.

Species	Type	Repbase TEs Length (Bp)	% in genome	TE proteins Length (Bp)	% in genome	De novo Length (Bp)	% in genome	Combined TEs Length (Bp)	% in genome
Tpse	DNA	790,274	1.15	391,929	0.57	1,583,996	2.30	2,311,148	3.35
	LINE	281,738	0.41	127,456	0.18	343,248	0.50	624,309	0.91
	SINE	4,329	0.01	0	0.00	351	0.00	4,680	0.01
	LTR	557,848	0.81	1,062,770	1.54	14,868,008	21.58	15,315,808	22.23
	Other	3,632	0.01	0	0.00	0	0.00	3,632	0.01
	Unknown	0	0.00	0	0.00	9,225,180	13.39	9,225,180	13.39
	Total	1,349,018	1.96	1,570,580	2.28	25,132,811	36.48	27,760,911	40.28
Tspi	DNA	723,547	1.14	577,090	0.91	2,675,255	4.21	3,201,127	5.04
	LINE	235,595	0.37	76,697	0.12	147,256	0.23	368,901	0.58
	SINE	4,213	0.01	0	0.00	27,945	0.04	31,330	0.05
	LTR	570,082	0.90	1,209,756	1.90	4,025,193	6.34	4,333,254	6.82
	Other	1,994	0.00	0	0.00	0	0.00	1,994	0.00
	Unknown	0	0.00	0	0.00	5,875,516	9.25	5,875,516	9.25
	Total	1,339,101	2.11	1,856,354	2.92	12,026,012	18.93	12,700,802	20.66

Supplementary Table 9. Expanded gene families contained unitary functional domain based on IPR annotations in *T. pseudospiralis* and *T. spiralis*. The numbers in second and third columns represent numbers of genes from a particular species under corresponding functional domain annotations. A chi-square test was applied to the pairwise comparison of numbers of genes within expanded gene families that containing TEs in *T. pseudospiralis* and *T. spiralis*, respectively. Gene families showed significant divergence between the two species ($p < 0.05$) were used for selection of unitary functional families.

Category	Tpse	Tspi	Function
Tpse expanded gene families	61	18	IPR004345; TB2/DP1/HVA22-related protein;
	13	7	IPR010987; Glutathione S-transferase, C-terminal;
	18	1	IPR006811; RNA polymerase II subunit A;
	11	5	IPR009072; Histone-fold; IPR001951; Histone H4;
Tspi expanded gene families	43	133	IPR004947; Deoxyribonuclease II;
	22	103	IPR005312; Protein of unknown function DUF1759;
	27	83	IPR001878; Zinc finger, CCHC-type;
	21	63	IPR000477; Reverse transcriptase domain;
	26	69	IPR008042; Retrotransposon, Pao;
	46	92	IPR029526; PiggyBac transposable element-derived protein;
	0	15	IPR008906; HAT dimerisation domain, C-terminal;
	18	47	IPR003100; PAZ domain;
	59	86	IPR009072; Histone-fold; IPR000558; Histone H2B;
	37	58	IPR004088; K Homology domain, type 1;
	27	45	IPR003609; PAN/Apple domain;
	0	4	IPR000511; Cytochrome c/c1 haem-lyase;
	146	160	IPR016024; Armadillo-type fold;
	5	7	IPR004875; DDE superfamily endonuclease, CENP-B-like;

Supplementary Table 10. Commonly and specifically orthologous groups between *T. pseudospiralis* and *T. spiralis* DNase II genes. Numbers in third and fourth columns represent genes from a particular species under corresponding orthologous groups.

	Orthologous groups	Tspi (Gene numbers)	Tpse (Gene numbers)
Common Groups (19)	254	13	2
	409	10	5
	939	9	1
	941	8	2
	1828	6	2
	1829	7	1
	3641	1	1
	8607	2	1
	8746	2	1
	11302	1	1
	11342	1	1
	11402	1	1
	11403	1	1
	11418	1	1
	12694	1	1
	12695	1	1
	12846	1	1
	13060	1	1
	13230	1	1
Specific Groups (7)	299	18	NA
	1831	8	NA
	8859	1	NA
	11159	2	NA
	11205	2	NA
	11329	2	NA
	11838	2	NA

Supplementary Table 11. Structural features of the C-type lectin domain models of *T. pseudospiralis* exhibit homology with host mammal lectin domains. The structural features are predicted by I-TASSER (https://zhanglab.ccmb.med.umich.edu/I-TASSER/). Ligand bingding site are predicted using COFACTOR and COACH. Template represents the proteins structurally close to the target protein in the PDB library (identified by TM-align).

Gene	Ca^{2+}	Putative coordinators	motif	Sugar	Putative sugar bingding residues	Template
TP10242	1	48,50,54,137	##	MMA	32,36,75,83,128	5xtsA
TP12499	1	40,42,46,128	##	GAL	90,92,94,98,115,116,117	
				GAL	59,102,104,115,117	4kzvA
				MMA	24,28,67,76,119	
TP02221	1	32,36,75,83	WIG	MMA	67,97,99,100,124,125	5vybA

MMA, methy alpha-D-mannopyranoside; GAL, beta-D-galactos

Supplementary Table 12. Gene repertoire of 6 differentially expressed species-specific SCOs between *T. pseudospiralis* and *T. spiralis* E/S proteins.

Gene	Expression level						Function
	Tpse			Tspi			
	Ad	ML	NBL	Ad	ML	NBL	
TP12446	88.1	142.7	86.1	19.2	34.9	35.0	E3 ubiquitin-protein ligase RNF128
TP09895	175.1	283.1	1337.9	102.1	87.6	795.2	Neuroendocrine 7B2 precursor
TP12523	68.4	112.5	16.7	18.7	13.8	8.5	palmitoyl-protein thioesterase
TP11614	140.3	96.4	46.4	26.9	33.7	79.9	Carboxylesterase
TP00267	51.2	79.7	120.3	24.8	28.1	19.6	Collagen beta-1,O-galactosyltransferase
TP06492	34.9	12.1	1.3	66.3	36.9	1.7	Gamma interferon inducible lysosomal thiol reductase
