# Supplementary material for: Comparative multi-omics analyses reveal differential expression of key genes relevant for parasitism between non-encapsulated and encapsulated Trichinella
Source: Commun Biol. 2021 Jan 29;4:134. doi: 10.1038/s42003-021-01650-z (PMC7846577; doi:10.1038/s42003-021-01650-z)
Supplement: Supplementary file 3 — Description of Supplementary Files [file 42003_2021_1650_MOESM3_ESM.pdf]

**Description of Additional Supplementary Files**

**File name: Supplementary Dataset 1**

**Description:** Potential parasitism-related functional proteins in *T. pseudospiralis* and *T. spiralis* genome. This table lists proteinases, GPCRs, protein kinases and excretory/secretory (E/S) proteins, as well as the candidate molecular targets for treatment of trichinellosis. The plus sign represents this gene shows homology against known drug target databases without homology with the host proteomes and the minus sign indicates this gene is not a drug target. Numbers in the brackets represent number of genes in corresponding category.

**File name: Supplementary Dataset 2**

**Description:** Bed file of methylation levels in genome of *T. pseudospiralis* in Ad, stage (≥ 5x read depth).

**File name: Supplementary Dataset 3**

**Description:** Bed file of methylation levels in genome of *T. pseudospiralis* in ML stage (≥ 5x read depth).

**File name: Supplementary Dataset 4**

**Description:** Bed file of methylation levels in genome of *T. spiralis* in Ad stage (≥ 5x read depth).

**File name: Supplementary Dataset 5**

**Description:** Bed file of methylation levels in genome of *T. spiralis* in ML stage (≥ 5x read depth).

**File name: Supplementary Dataset 6**

**Description:** Expression levels of each gene in three life stages (Ad, ML and NBL) of *T. pseudospiralis*.

**File name: Supplementary Dataset 7**

**Description:** Orthologous groups generated by Markov Clustering in the genomes of parasitic nematode *T. pseudospiralis* (Tpse), *T. spiralis* (Tspi), *C. elegans* (Cele), *M. incognita* (Minc), *B.*

*malayi* (Bmal), with *D. melanogaster* (Dmel) serving as the outgroup. This table lists numbers of orthologous gene families generated by Orthomcl and numbers of genes from each species for a particular combination of species. Expansion and contraction events are also listed. For instance, Tpse-expanded represents *T. pseudospiralis* displayed an expansion event under corresponding gene family.

**File name: Supplementary Dataset 8**

**Description:** Expression levels of each gene in three life stages (Ad, ML and NBL) of *T. spiralis*.

**File name: Supplementary Dataset 9**

**Description:** Comparison of E/S proteins in *T. pseudospiralis* and *T. spiralis* genomes.
