# Supplementary material for: Comparative multi-omics analyses reveal differential expression of key genes relevant for parasitism between non-encapsulated and encapsulated Trichinella
Source: Commun Biol. 2021 Jan 29;4:134. doi: 10.1038/s42003-021-01650-z (PMC7846577; doi:10.1038/s42003-021-01650-z)
Supplement: Supplementary file 13 — Reporting Summary [file 42003_2021_1650_MOESM13_ESM.pdf]

nature research

Corresponding author(s): Fei Gao

Last updated by author(s): Dec 1, 2020

Reporting Summary

Nature Research wishes to improve the reproducibility of the work that we publish. This form provides structure for consistency and transparency in reporting. For further information on Nature Research policies, see our Editorial Policies and the Editorial Policy Checklist.

Statistics

For all statistical analyses, confirm that the following items are present in the figure legend, table legend, main text, or Methods section.

n/a	Confirmed	
☐	☒	The exact sample size (*n*) for each experimental group/condition, given as a discrete number and unit of measurement
☐	☒	A statement on whether measurements were taken from distinct samples or whether the same sample was measured repeatedly
☐	☒	The statistical test(s) used AND whether they are one- or two-sided<br>*Only common tests should be described solely by name; describe more complex techniques in the Methods section.*
☒	☐	A description of all covariates tested
☐	☒	A description of any assumptions or corrections, such as tests of normality and adjustment for multiple comparisons
☐	☒	A full description of the statistical parameters including central tendency (e.g. means) or other basic estimates (e.g. regression coefficient) AND variation (e.g. standard deviation) or associated estimates of uncertainty (e.g. confidence intervals)
☐	☒	For null hypothesis testing, the test statistic (e.g. *F*, *t*, *r*) with confidence intervals, effect sizes, degrees of freedom and *P* value noted<br>*Give P values as exact values whenever suitable.*
☒	☐	For Bayesian analysis, information on the choice of priors and Markov chain Monte Carlo settings
☒	☐	For hierarchical and complex designs, identification of the appropriate level for tests and full reporting of outcomes
☒	☐	Estimates of effect sizes (e.g. Cohen's *d*, Pearson's *r*), indicating how they were calculated

Our web collection on statistics for biologists contains articles on many of the points above.

Software and code

Policy information about availability of computer code

Data collection	Illumina Hiseq system for generation of next sequencing reads for both DNA and RNA.<br>PacBio RS II platform for generation of Pacbio SMRT sequencing data.
Data analysis	Genome assembly for next generation sequencing reads: Soapdenovo (v2.04);<br>Genome assembly for Pacbio SMRT sequencing data: Canu;<br>Analysis of methylation data: BSMAP (v2.73);<br>Analysis of RNA-seq data: TopHat (v2.0.12) and Cufflinks (v2.2.1);<br>Analysis of gene family expansion and contraction events: OrthoMCL;

For manuscripts utilizing custom algorithms or software that are central to the research but not yet described in published literature, software must be made available to editors and reviewers. We strongly encourage code deposition in a community repository (e.g. GitHub). See the Nature Research guidelines for submitting code & software for further information.

Data

Policy information about availability of data

All manuscripts must include a data availability statement. This statement should provide the following information, where applicable:

- Accession codes, unique identifiers, or web links for publicly available datasets
- A list of figures that have associated raw data
- A description of any restrictions on data availability

Raw genome sequencing data were deposited in the National Center for Biotechnology Information with the following accession number: SAMN08905168 under project PRJNA451013; SRP140458 for transcriptome and WGBS data; The genome sequence has also been deposited at DDBJ/ENA/GenBank (accession number QAWF00000000).

Field-specific reporting

Please select the one below that is the best fit for your research. If you are not sure, read the appropriate sections before making your selection.

☒ Life sciences ☐ Behavioural & social sciences ☐ Ecological, evolutionary & environmental sciences

For a reference copy of the document with all sections, see nature.com/documents/nr-reporting-summary-flat.pdf

Life sciences study design

All studies must disclose on these points even when the disclosure is negative.

Sample size	Trichinella pseudospiralis (ISS13) collected from muscle larvae stage was used for library construction of NGS and Pacbio SMRT sequencing for genome assembly. DNA samples collected from Ad, ML and NBL stages were used for WGBS analysis. RNA samples collected from Ad, ML and NBL stages were used for RNA-seq analysis.
Data exclusions	No data has been excluded.
Replication	The detection of enzymatic activity of DNMTs, transfection efficiency of TP12446 into lentivirus vector, and immune-fluorescence analysis of C2C12 myoblasts were conducted with at least two biologically independent replicates.
Randomization	Randomization was not relevant to this study.
Blinding	The first author was entireley blinded on all the smaples that were measured. The blingding of the samples was conducted by the senior author.

Reporting for specific materials, systems and methods

We require information from authors about some types of materials, experimental systems and methods used in many studies. Here, indicate whether each material, system or method listed is relevant to your study. If you are not sure if a list item applies to your research, read the appropriate section before selecting a response.

Materials & experimental systems

n/a	Involved in the study	
☐	☒	Antibodies
☐	☒	Eukaryotic cell lines
☒	☐	Palaeontology and archaeology
☐	☒	Animals and other organisms
☒	☐	Human research participants
☒	☐	Clinical data
☒	☐	Dual use research of concern

Methods

n/a	Involved in the study	
☒	☐	ChIP-seq
☒	☐	Flow cytometry
☒	☐	MRI-based neuroimaging

Antibodies

Antibodies used	Monoclonal mouse anti-myosin heavy chain (MYH1/2/3, Santa Cruz Biotechnology, USA);<br>Secondary antibody (Goat Anti-Rabbit IgG H&L, Alexa Fluor® 555, ab150078).
Validation	Monoclonal mouse anti-myosin heavy chain (MYH1/2/3, Santa Cruz Biotechnology, USA);<br>Secondary antibody (Goat Anti-Rabbit IgG H&L, Alexa Fluor® 555, ab150078).

Eukaryotic cell lines

Policy information about cell lines

Cell line source(s)	C2C12 skeletal muscle cells were obtained from American Type Culture Collection (ATCC, Manassas, VA).
Authentication	Cells were cultured at 37 ˚C for 48 h under 5% CO2 in Dulbecco's modified Eagle's medium-high glucose (DMEM, GIBCO, USA) supplemented with 10% (v/v) fetal bovine serum (FBS, GIBCO, USA), 100 U/ml penicillin and 100 μg/ml streptomycin. For differentiation into myotubes, cells were grown to 90% confluency. Cells was then switched to differentiation media at 37 ˚C for 4~5 d under 5% CO2 in DMEM supplemented with 5% (v/v) horse serum (HS, GIBCO, USA), 100 U/ml penicillin and 100 μg/ml streptomycin
Mycoplasma contamination	The C2C12 cell lines were tested negative for mycoplasma contamination.
Commonly misidentified lines (See ICLAC register)	n/a

Animals and other organisms

Policy information about studies involving animals; ARRIVE guidelines recommended for reporting animal research

Laboratory animals	Trichinella pseudospiralis (ISS13); Female BALB/c rats
Wild animals	The study did not involve wild animals.
Field-collected samples	The Trichinella samples were produced in female BALB/c rats. Larvae were recovered from muscle tissue of infected rats on 35 days post infection (dpi) by artificial digestion with pepsin-HCl (1% pepsin, 1% HCl at 42˚C for 45 min). Adult worms and newborn larvae were isolated from experimentally infected rats orally inoculated with T. pseudospiralis with a single dose of 8,000 larvae per rat.
Ethics oversight	All experimental protocol involving animals have been reviewed and approved by the Ethical Committee of the Jilin University affiliated with the Provincial Animal Health Committee, Jilin Province, China (Ethical Clearance number IZ-2009-08).

Note that full information on the approval of the study protocol must also be provided in the manuscript.
